# The landscape of cyclin-dependent kinase 4/6 inhibitors in solid malignancies: emphasis on immunotherapy combinatorial strategies

**DOI:** 10.1007/s12032-025-02996-8

**Published:** 2025-08-26

**Authors:** Sara A. Hussein, Ahmed H. Saadawy, Eman Badr, Maha R. A. Abdollah, Neamtullah Wael, Rasha M. Allam, Ahmed M. Al-Abd, Ana Maria Roncero Sanchez, Mai F. Tolba

**Affiliations:** 1 Department of Pharmacology and Toxicology, Faculty of Pharmacy, Ain Shams University, Abbassia, 11566 Cairo Egypt; 2 Center for Drug Discovery Research and Development, Ain Shams University, Cairo, Egypt; 3 Department of Biological Sciences, Dornsife College of Letters, Arts and Sciences, University of Southern California, Los Angeles, CA USA; 4 Biomedical Sciences Program, University of Science and Technology, Zewail City, Giza, 12578 Egypt; 5 Faculty of Computers and Artificial Intelligence, Cairo University, Giza, 12613 Egypt; 6 Department of Pharmacology, Faculty of Pharmacy, The British University in Egypt, El Sherouk City, Cairo, Egypt; 7 The Health Research Center of Excellence; Drug Research and Development Group, The British University in Egypt, El Sherouk City, Cairo, Egypt; 8 Department of Pharmacology, Medical and Clinical Research Institute, National Research Centre, Cairo, Egypt; 9 CNIO-Spanish National Cancer Research Center, Melchor Fernández Almagro, Madrid, Spain

**Keywords:** Immunotherapy, Immune checkpoint inhibitors, CDK4/6 inhibitors, Clinical trials, Solid tumors

## Abstract

Given the pivotal role of CDK4/6 deregulation in the pathogenesis of various malignancies, CDK4/6 inhibitors have been incorporated into cancer treatment protocols. Beyond their antiproliferative effects, these inhibitors exhibit prominent immunomodulatory properties. Preclinical data indicated that CDK4/6 inhibitors enhance the tumor cell antigen presentation capacity and restrict the proliferation of immunosuppressive regulatory T-cells. Furthermore, studies showed that CDK4/6 inhibitors could enhance the development of memory T-cell in vivo in melanoma mouse models and in the clinical setting in breast cancer (BC) patients. In silico analysis identified 225 protein targets for CDK4/6 inhibitors involved in 57 immune-related pathways which further supported the immune-modulatory potential of these inhibitors in innate immunity, cytokine signaling, and adaptive immunity. Furthermore, 24 targets exhibited significantly higher protein expression in immune cell types compared to non-immune cell types, as indicated by data from the Human Protein Atlas. The favorable modulation of T-cell immunity by CDK4/6 inhibitors could complement the antitumor effect of immunotherapies, such as immune checkpoint inhibitors (ICIs), which hamper PD-1 /PD-L1 signaling and result in the reactivation of the antitumor T-cell response. Herein, we explore the clinical portfolio of CDK4/6 inhibitors across various solid malignancies, with emphasis on emerging combinations with ICIs.

**Supplementary Information:**

The online version contains supplementary material available at 10.1007/s12032-025-02996-8.

## Introduction

**Fig. 1:**
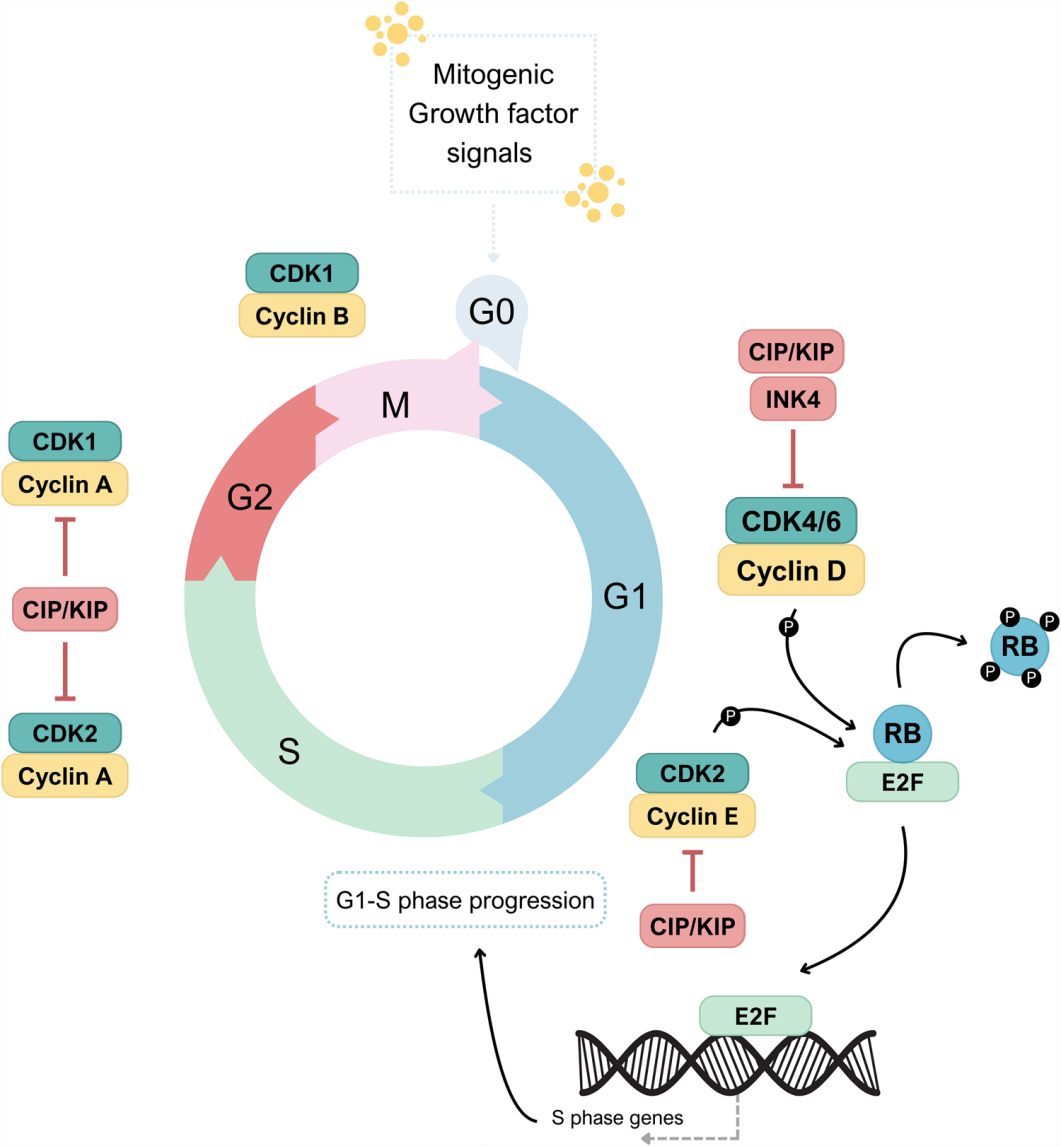
Mechanism of cell cycle regulation by Cyclin D–CDK4/6-Rb-E2F axis

Cyclin-dependent kinases (CDKs) are essential for controlling the progression of the cell cycle, and their activities are modulated by cyclins (which act as activators) or cyclin-dependent kinase inhibitors (CKIs) [1–3]. CDK4 and CDK6 (CDK4/6) are particularly critical  due to their fundamental role in the G1/S transition as depicted in Fig. 1 [1]. CDKs are a family of serine/threonine protein kinases, which form heterodimers with their respective regulatory cyclin subunits and phosphorylate specific substrates. In the G1 phase, the substrate for CDK4/6 is retinoblastoma susceptibility protein (Rb), which interacts with the E2F transcriptional family in its hypophosphorylated state, whereby it suppresses the transcription of target genes, and when a cell senses mitogenic signals, CDK4/6 are activated by cyclin D and then phosphorylate Rb, thus relieving the inhibition [2, 3]. Sequential phosphorylation of Rb by CDK4/6 and CDK2 results in E2F activation and transcription initiation of genes required for S-phase progression [1]. Deregulation of cell cycle is a hallmark of cancer. Aberrant activity of cyclin D–CDK4/6 pathway has been described in many types of cancer and it invariably leads to uncontrolled cell proliferation with rapid progression through the cell cycle [4–6]. Because of their key role in the cell cycle regulation and implication in carcinogenesis, CDK4/6 inhibition represents an attractive intervention for cancer management as they prevent the phosphorylation of Rb leading to halting the cell cycle progression [7, 8]. Cyclin-D 1 protein is often overexpressed in 50% of patients with breast cancer [9]. Since CDK4/6 are crucial checkpoints for G1/S transition, specific CDK4/6 inhibitors were approved to tackle these kinases [10]. Abemaciclib, palbociclib, and ribociclib are three oral CDK4/6 inhibitors that successfully secured FDA approval for advanced breast cancer (BC) patients with hormone receptor-positive (HR +) and human epidermal growth factor receptor 2-negative (HER2-) tumor status[9]. Palbociclib and ribociclib were both developed from pyrido [2,3-d] pyrimidin-7-one scaffold. However, abemaciclib was developed from 2-anilino-2,4-pyrimidine-[5-benzimidazole] scaffold [11]. The three inhibitors act on CDK4 and 6. However, abemeciclib inhibits a wider scope of kinases including CDK1,2, 5, 9, 14, 16–18 along with GSK3α/β and PIM1 kinases [11]. The development of  CDK4/6 inhibitors represents a key milestone in cancer therapeutics, demonstrating improved overall survival with manageable toxicities [9].

It is noteworthy that CDK4/6 inhibitors have the capacity to act beyond the cell cycle through the modulation of cellular energetics and metabolic reprogramming [12]. Studies also reported that CDK4/6 inhibitors can induce senescence, autophagy [13], as well as epigenetic modifications [14]. Interestingly CDK4/6 inhibitors were also reported to exhibit a positive impact within the tumor immune microenvironment by augmenting the antitumor immunity [8].

**Fig. 2:**
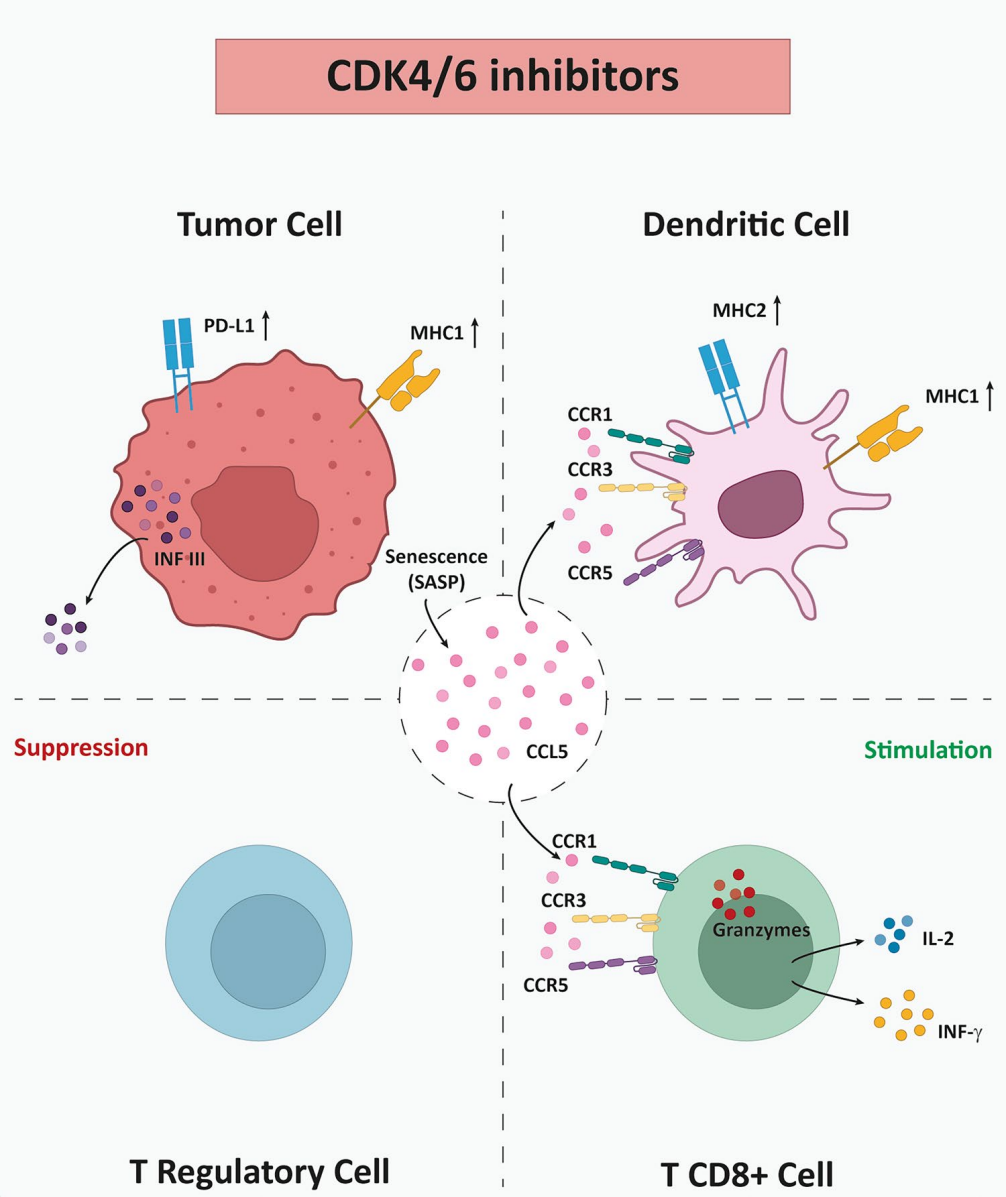
CDK4/6 inhibitors possess favorable immunomodulatory effects within the tumor microenvironment (TME)

Studies supported that CDK4/6 inhibitors enhance tumor antigen presentation by macrophages and dendritic cells [8]. Furthermore, they promote inflammatory signaling in tumor cells, stimulate immunogenic cell death, reduce immunosuppressive cell populations (Tregs and myeloid-derived suppressor cells MDSCs). Additionally, they are capable of activating effector T-cells and boost the infiltration of B-lymphocytes and natural killer (NK) cells [15]. The immune-modulatory effects of CDK4/6 inhibitors are depicted in Fig. 2. In this review, we highlight the ongoing clinical studies that explore the development of combinations of CDK4/6 inhibitors with PD-1/PD-L1 immune checkpoint immunotherapies in solid malignancies (Table 1).

**Table 1:** The current clinical trials investigating CDK4/6 inhibitor/Immunotherapy combinations in solid tumors

Cancer Type	Disease subtype/stage	Drug Combination	Trial Phase	Main Outcomes	Key Toxicities	Ref
Breast Cancer	HR + /HER2- metastatic breast cancer	Abemaciclib + Pembrolizumab ± Anastrozole	Phase Ib	ORR 23.1%-28.6%; DCR 82.1–84.6%	Grade ≥ 3 neutropenia, diarrhea, hepatotoxicity; 2 deaths	[30], NCT02779751
	HER2 − MBC and metastatic ovarian cancer	Ribociclib + Spartalizumab + Fulvestrant	Phase Ib	CBR 12.5%; PFS 3.1 mo; OS 6.0 mo in HER2-MBC	Grade ≥ 3 neutropenia (50%), liver enzyme elevation	[37], NCT03294694
	HR + , HER2- MBC	Abemaciclib + Nivolumab + Endocrine Therapy	Phase II	ORR 54.5% (fulv), 20% (letro); DCR 90.9% (fulv), 80% (letro)	Severe immune-related AEs; trial terminated	[31], JapicCTI-194782
	HR^+^ HER2^−^ MBC	Palbociclib + Avelumab + Fulvestrant	Phase II	Median PFS 8.1 mo vs 4.6 mo without avelumab	Neutropenia (grade 3 to 4), fatigue, anemia, and thrombocytopenia	[32, 33]
	Stage IV HR^+^ HER2^−^ MBC	Palbociclib + Pembrolizumab + Letrozole	Phase I/II	Median PFS 25.2 mo; Median OS 36.9 mo; well tolerated	Grade ≥ 3 neutropenia (83%) and leucopenia (65%)	[34]
	HR^+^ HER2^−^ 1ry BC	Palbociclib + Nivolumab + Anastrozole	Phase Ib/II	Trial terminated early	High prevalence of Grade 3/4 hepatotoxicity	[35], NCT04075604
HNSCC	Recurrent/ metastatic HNSCC	Ribociclib + Spartalizumab	Phase I	ORR 23%; PFS 2.1 mo	Tolerable	[50], NCT04213404
		Palbociclib + Avelumab + Cetuximab	Phase I	ORR by RECIST v1.1 was 42%	Well-tolerated with grade 1 to 2 treatment-related adverse events as rash and fatigue	NCT03498378
NSCLC	Stage IV NSCLC	Abemaciclib + Pembrolizumab	Phase Ib	ORR 24% (non-sq), 8% (sq); OS 6–27.8 mo	Grade ≥ 3 toxicities; trial terminated	[62], NCT02779751
Liver cancer	Hepatocellular carcinoma (HCC)	Abemaciclib + Nivolumab	Phase II	Trial terminated prematurely	Safety concerns like sepsis (14.29%)	NCT03781960
Ovarian Cancer	Metastatic ovarian cancer	Ribociclib + Spartalizumab + Fulvestrant	Phase Ib	PR (13.3%)	Hypertransaminasemia,neutropenia, fatigue, and anemia	[37]
Melanoma	Metastatic Cutaneous Melanoma	Abemaciclib + Anti-PD-L1 (LY3300054)	Phase Ia/Ib	1 PR (5%), 10 SD (50%) in concurrent arm	Diarrhea, fatigue; autoimmune hepatitis in lead-in arm	[73]
Prostate Cancer	Metastatic castration-resistant prostate cancer	Abemaciclib + Atezolizumab	Phase II	Ongoing	Awaiting results	[79], NCT04751929
Glioblastoma	Recurrent glioblastoma	Abemaciclib + Pembrolizumab	Phase II	Trial halted	Safety concerns	NCT04118036
CRC	Advanced CRC	SHR6390 + Camrelizumab	Phase Ib/II	Ongoing	Awaiting results	NCT03601598
Sarcomas	Soft tissue sarcoma	Palbociclib and pembrolizumab	Phase Ib	Ongoing	Awaiting results	NCT06113809
	advanced dedifferentiated liposarcoma	Palbociclib and retifanlimab	Phase II	Ongoing	Awaiting results	NCT04438824

Furthermore, we performed an in silico analysis that offers insights into the targets and pathways affected by the specific drug classes to provide more insights on their potential molecular targets. Herein, the protein targets of the four reviewed CDK4/6 inhibitors (abemaciclib, dalpiciclib, palbociclib, and ribociclib) were predicted, along with the biological pathways in which they are involved, as well as their cell-type-specific protein expression. The primary objective was to investigate the role of CDK4/6 inhibitors in immunological pathways for the purpose of gaining insights into their immune-modulatory role when administered in conjunction with immune checkpoint inhibitors. Overall, the data from this analysis provided further support for the favorable immune-modulatory role for CDK4/6 inhibitors, which could be exploited to boost the efficacy of ICIs and improve response rates in cancer patients.

## The clinically investigated ICI combinations with CDK4/6 inhibitors in solid tumors

### Breast cancer

CDK4/6 kinases are integral to the orchestration of cellular replication in the breast tissue [16, 17]. The upregulated activity of cyclin D–CDK4/6 axis in breast cancer (BC) highlights it as a legitimate therapeutic target [16–18]. Hormone receptor-positive (HR +) BC is more sensitive to CDK4/6 inhibitors given that estrogen boosts CCND1 gene expression which encodes cyclin, contributing to aberrant cell cycle [19–21]. CDK4/6 inhibitors combined with anti-estrogens were approved by the FDA for BC management especially in advanced-stage HR + /HER2- subtype [22–27]. Certain subsets of BC proved to be more enriched with tumor-infiltrating lymphocytes (TILs) [28]. The greater abundance of TILs was linked to better prognosis. [29]. The immune checkpoint PD-L1 is overexpressed in about 20% of BC patients. TNBC and HER2-positive subtypes are known to possess higher PD-L1 expression (33% and 56%) compared to ER + BC (11%) [29].

The combination of pembrolizumab (anti-PD-1 mAb) with chemotherapy has acquired FDA approval for managing inoperable or locally advanced/metastatic PD-L1-positive TNBC. Additionally, pembrolizumab is approved as a monotherapy for early-stage TNBC with high risk of recurrence and for treating metastatic or inoperable breast malignancies that are mismatch repair deficient (dMMR) or have high microsatellite instability (MSI-H) [30]. However, more efforts are needed to amplify the benefit of immunotherapy to a broad range of BC patients.

One of the promising avenues is the integration of CDK4/6 inhibitors with immune checkpoint blockade. The combination of abemaciclib/pembrolizumab ± anastrozole was tested in HR + /HER2- metastatic BC patients in a phase Ib trial (NCT02779751). The 1st cohort consisted of patients who were treatment-naïve to systemic anticancer therapy and received a triple combination of abemaciclib/pembrolizumab/ anastrozole. The 2nd cohort included patients previously treated with one or two lines of chemotherapy. They received abemaciclib/pembrolizumab without anastrazole. Despite that the disease control rate was a bit higher in cohort 1 (84.6 versus 82.1% in cohort 2). The overall response rate (ORR) was much higher in cohort 2 (28.6% vs 23.1% in cohort 1). [30]. Assessment of the safety data showed that cohort 1 experienced an increased prevalence of grade ≥ 3 adverse events (AEs) including neutropenia, diarrhea, and severe alterations in liver function tests. It is noteworthy that treatment-induced AEs resulted in 2 deaths in cohort 1. Patients in both cohorts showed higher incidence of pneumonitis and interstitial lung disease. Given the high incidence of toxicity, the benefit/risk analysis did not advocate further testing of the abemaciclib/pembrolizumab combination for HR + /HER2 − metastatic BC.

Abemaciclib was tested in combination with a different checkpoint inhibitor (nivolumab) and endocrine therapy (fulvestrant or letrozole) through a phase II trial in the same setting of HR + /HER2- metastatic BC [31]. The triple combination showed clinical activity with an ORR of 54.5% with fulvestrant vs. 20% letrozole endocrine therapy. Similarly, the disease control rate (DCR) was higher with fulvestrant vs. letrozole (90.9% vs 80.0%). Despite the clinical activity of the tested combination, the development of severe and sustained immune-related AEs led to study termination. Findings from the two clinical studies imply that combining abemaciclib with immunotherapy may amplify treatment-related toxicities.

Various clinical trials tested the therapeutic benefit of palbociclib combinations with immunotherapy (avelumab/ nivolumab/ pembrolizumab) for breast cancer. Two palbociclib combinations were promising in HR + /HER2- metastatic BC. The randomized phase II trial PACE tested palbociclib/avelumab/fulvestrant combination for HR + /HER2- metastatic BC previously progressed on CDK4/6 inhibitor + aromatase dual therapy. Adding avelumab extended the median PFS to 8.1 vs. 4.6 months without avelumab which encourages further investigation of this triple combination in this population [32, 33]. Palbociclib was investigated in combination with pembrolizumab/letrozole as a front-line therapy in a phase I/II clinical trial for patients with stage-IV HR + /HER2- metastatic BC [34]. The data generated from the trial indicated significant clinical benefits, with 31% of patients achieving a complete response, 25% experiencing a partial recovery, and 31% maintaining a stable disease. The median PFS was 25.2 months, and the median OS was 36.9 months. The combination is tolerable, and grade ≥ 3 AEs included neutropenia (83%) and leucopenia (65%). Additional research is warranted to elucidate the immune-modulatory mechanisms of palbociclib in this therapeutic context [34]. On the other hand, testing palbociclib in combination with nivolumab/ anastrozole in ER + /HER2- primary BC showed disappointing outcomes in the CheckMate 7A8 trial. The data of the early run-in-phase showed a high prevalence of grade 3/4 hepatotoxicity. Therefore, the study was terminated [35].

PAveMenT phase Ib trial (NCT04360941) investigated palbociclib/ avelumab combination in patients with androgen receptor-positive (AR +) TNBC(*n =* 12) and ER + /HER2- advanced BC(*n =* 2) [36]. The dose-finding phase investigated two dosing schedules for palbociclib including intermittent dosing (75–125 mg) versus continuous dosing (100 mg). Four out of twelve TNBC patients were AR + , and five out of ten TNBC patients with an adequate amount of tissue samples positive for PD-L1 expression. Notably, one of the PD-L1 + ve patients and, separately, two of the five PD-L1 − ve TNBC cases were AR + . The outcomes showed that all the tested schedules were tolerable. However, the continuous dosing (100 mg) was recommended for the anticipated expansion phase in AR + TNBC because of the rapid proliferation characteristics of TNBC and the greater potential for tumor re-growth with intermittent dosing (NCT04360941).

A different triple combination (Ribociclib/spartalizumab ± fulvestrant) was assessed clinically for HR + /metastatic BC and metastatic ovarian cancer in a phase Ib trial [37]. The clinical outcomes were variable. One breast cancer patient accomplished a partial response (ORR = 6.25%), while another had stable disease lasting over 24 weeks. The clinical benefit rate (CBR) for the group was 12.5% through a median follow-up period of 3.5 months. Notably, both patients who experienced clinical benefit received the triple combination therapy. The median PFS across all patients was 3.1 months, with a median overall survival (OS) of 6.0 months. The trial also highlighted that grade ≥ 3 AEs included neutropenia (50%) and elevated liver transaminases (25%). Despite that the combination of ribociclib/spartalizumab was deemed tolerable with AEs comparable to those observed with monotherapy. Further dose expansion cohorts are currently recruiting patients with HR + metastatic BC and metastatic ovarian cancer [37].

Although trials involving abemaciclib in combination with immunotherapy were discontinued due to heightened toxicity concerns, investigations into palbociclib-based immunotherapy regimens have yielded encouraging outcomes. Similarly, early data from studies evaluating ribociclib with spartalizumab suggest potential therapeutic promise.

### Head and neck squamous cell carcinoma (HNSCC)

HNSCC is well suited for ICIs due to abundant evidence demonstrating immune evasion mechanisms that contribute to disease progression. These mechanisms often involve the PD-1/PD-L1 axis. Clinical trials showed the benefit of ICIs (nivolumab and pembrolizumab) in recurrent/metastatic HNSCC patients with better PFS and OS compared to standard therapy (NCT01848834; NCT02358031; NCT02252042; NCT02105636) [38–41]. Therefore, ICIs received FDA approval as a first-line treatment for metastatic and recurrent HNSCC [42]. CCND1 amplification and CDKN2A mutations, and Rb presence are considered as biomarkers to predict the activity of CDK4/6 inhibitors in HPV-negative HNSCC [43–45]. Preclinical studies proved the positive effects of CDK4/6 inhibitors in HNSCC. Abemaciclib suppressed the growth of HNSCC cell lines (OSC-19, FaDu, and YD-10B) and led to cell cycle arrest at G0/G1. The same study also indicated that abemaciclib significantly suppressed the growth of OSC-19 xenografts in mice [46]. Similarly, ribociclib exhibited cytostatic effect in HPV-negative cell lines (Detroit562, Cal27, FaDu, SCC9, SCC15, and SCC25) [47]. In vivo data indicated that ribociclib reduced tumor growth in HNSCC xenograft in mice [48].

Clinical studies specifically focus on HPV-negative patients due to the presence of overactive CDK4/6-Rb pathway; in contrast, HPV-positive HNSCC is characterized by the viral oncoprotein E7, which degrades Rb protein, so the upstream CDK4/6 pathway rendered redundant, making HPV-positive patients resistant to CDK4/6 inhibitors[49]. From an immunotherapy perspective, HPV-positive tumors are typically more immunogenic and demonstrate higher response rates to ICIs immunotherapy. Therefore, clinical studies regarding combination therapy of CDK4/6 inhibitors and ICIs target the HPV-negative patient because they respond well to CDK4/6 inhibitors. Additionally, they can augment their modest response to ICIs [50].

The combination of ribociclib/spartalizumab was examined in a phase I trial in patients with recurrent/metastatic HPV-negative HNSCC [51]. The efficacy was modest with ORR 23% and median PFS of 2.1 months. The safety data showed that the combination is tolerable. On the other hand, a phase I study investigated abemaciclib/ nivolumab combination in patients with recurrent/metastatic HNSCC patients who recurred or progressed within 6 months after platinum-based chemotherapy (NCT03655444). The study was terminated due to the development of severe treatment-related toxicity [52]. Other ongoing clinical trials for ICI and CDK4/6 inhibitors in patients with recurrent/metastatic HNSCC include the combination abemaciclib/pembrolizumab (NCT03938337) and palbociclib/avelumab/cetuximab (NCT03498378).

### Lung cancer (non-small cell lung cancer NSCLC)

Cell cycle deregulation and disruption of the CDK/cyclin/Rb pathway are adversely implicated in the pathology of NSCLC [53]. Several clinical trials tested CDK4/6 inhibitors in patients with advanced-stage NSCLC (NCT02154490; NCT01291017; NCT02152631; NCT02079636; NCT01394016; and NCT02411591). Abemaciclib demonstrated better PFS and ORR in a phase III trial concerning patients with stage-IV NSCLC [54]. Additionally, palbociclib exhibited antitumor activity in a phase II study targeting CDKN2A-mutant NSCLC, yielding a median PFS of 8.1 weeks and a median OS of 21.6 weeks [55].

PD-L1 is expressed in 35–95% of NSCLC patients [56], and ICIs are currently approved by the FDA for advanced pretreated NSCLC [57, 58]. Nivolumab showed longer PFS, and OS versus docetaxel in CheckMate 017 and CheckMate 057 clinical trials [59, 60]. A pooled analysis of CheckMate 017 and CheckMate 057 studies revealed that the two-year OS rates with nivolumab compared to docetaxel were 23% versus 8% in squamous NSCLC and 29% versus 16% in non-squamous (NS)-NSCLC [61]. The combination of Abemaciclib/pembrolizumab was evaluated in a phase Ib trial in stage-IV NSCLC patients (NCT02779751) [62]. The study enrolled two cohorts: cohort 1 comprising patients with non-squamous non-small cell lung cancer (NS-NSCLC), and cohort 2 consisting of squamous NSCLC cases. Partial responses were observed in 24% of cohort 1 and 8% of cohort 2. Disease control rates were 56% and 64%, respectively, with median PFS of 7.6 months in cohort 1 and 3.3 months in cohort 2, and median OS of 27.8 and 6.0 months. Despite these promising clinical outcomes, both cohorts experienced a high incidence of grade ≥ 3 adverse events, ultimately leading to trial discontinuation. Interestingly, the same therapeutic combination demonstrated a more favorable safety profile in patients with HR + /HER2 − BC (NCT02079636)[63].

### Hepatocellular carcinoma (HCC)

Perturbations of cyclin D-CDK4/6/Rb signaling are frequently reported in HCC [64]. CDK4/6 inhibitors proved to be efficacious for combination with tyrosine kinase inhibitors in the management of HCC [65, 66]. Clinically, palbociclib was tested in a phase II trial in adult patients with advanced HCC who have proved resistant/intolerant of first-line sorafenib. Results showed that palbociclib was well tolerated and has activity against advanced HCC with median OS of 19 weeks, and median time to progression (TTP) of 24 weeks. Safety reports indicated that grade ≥ 3 AEs included neutropenia and thrombocytopenia (NCT01356628) [67].

ICIs demonstrated clinical benefits in patients with advanced HCC. The phase I/II trial CheckMate 040 investigated nivolumab in patients with advanced HCC and Child–Pugh B cirrhosis. Data generated from this study demonstrate clinical efficacy of nivolumab (55% disease control rate) with an acceptable safety profile in patients with HCC with Child–Pugh B status who have mild to moderate impairment of liver function or liver decompensation that limits other treatment options. Furthermore, the efficacy of nivolumab was compared to sorafenib in the multicenter phase III trial CheckMate 459 [68]. Efficacy data indicated that nivolumab was superior to sorafenib in ORR (15% vs 7%) and the median OS (16.4 vs 14.7 months). Nivolumab was well tolerated in patients with advanced HCC. The efficacy of pembrolizumab in advanced-stage pretreated HCC patients was assessed in a phase III trial (KEYNOTE-240) [69]. The results showed that pembrolizumab mitigated the risk of death by 22% and improved PFS compared to placebo in patients with advanced HCC. The ORR was 16.9% in pembrolizumab arm versus 2.2% in placebo arm. The combination of abemaciclib/nivolumab was examined in a phase II clinical trial. However, the study was concluded prematurely due to safety concerns (NCT03781960).

### Ovarian cancer

A few preclinical studies investigated co-treatment with ICIs and CDK4/6 inhibitors in ovarian cancer. Zhang et al. studied the combination of abemaciclib/anti-PD-1 antibody in a murine syngeneic ovarian cancer model [70]. Abemaciclib-alone augmented a pro-inflammatory tumor response by increasing the tumor infiltration of immune cells, particularly B-cells and cytotoxic T-cells. While the combination therapy significantly increased the activity of B-cells and cytotoxic T-cells leading to a more profound pro-inflammatory anti-tumor response and consequently a synergistic reduction in tumor volume [70]. Overall, this study proved that the combined treatment strategy of CDK4/6 and PD-1 inhibitors holds great promise and offers potential for treating poorly immune-infiltrated ovarian cancers. There is an ongoing phase Ib clinical trial investigating the combination of ribociclib/spartalizumab ± fulvestrant for patients with HR + metastatic BC or metastatic ovarian cancer [37]. The efficacy data for the ovarian cancer cohort are still anticipated.

### Melanoma

Immunotherapies have become central for the treatment of advanced melanoma [71]. Nonetheless, a modest set of studies has combined ICIs with CDK4/6 inhibitors especially after the emergence of refractory cases despite the initial success of immunotherapies in advanced melanoma [72]. Abemaciclib was investigated in combination with anti-PD-L1 antibody (LY3300054) in 24 patients as part of the PACT phase Ia/Ib clinical trial in patients with different types of solid malignancies including 1 melanoma patient [73]. Abemaciclib was given either first (lead-in) followed by LY3300054 in 4 patients, which triggered autoimmune hepatitis, or concurrently with LY3300054 (*n =* 20) which was better tolerated by patients. The most observed AEs in patients receiving the concurrent therapy were diarrhea (55%) and fatigue (35%). In the lead-in arm, 2 patients experienced stable disease, and one showed disease progression. However, in the concurrent arm, 1 patient had partial response, 10 patients had stable disease, and 6 patients had progressive disease [73]. The findings of this trial suggest that combination dosing strategies require better optimization to improve tolerability. This is illustrated by the adjustment from a lead-in to a concurrent dosing sequence in the combination of abemaciclib with LY3300054. Furthermore, the study provided preliminary evidence of the combination therapy*’*s durable clinical benefit, with some patients achieving either a partial response or stable disease [73]. However, more clinical studies are needed to test the combination in larger number of melanoma patients to validate its benefit.

### Prostate cancer

Similar to estrogen receptors, the androgen receptors regulate the transition from G1 phase to S-phase contributing to cell cycle control. Therefore, CDK4/6 inhibitors are capable of disrupting androgen receptors signaling [74, 75]. Abemaciclib showed cytotoxic and apoptotic activity in vitro against metastatic castration-resistant prostate cancer (mCRPC) androgen receptor (AR) negative PC-3 and AR mutant LNCaP PC cells [76]. Clinical testing of ribociclib in combination with docetaxel in a phase Ib/II clinical study in mCRPC showed clinical benefits observed by radiographic PFS (8.1 months) with acceptable toxicity (NCT02494921) [77]. Reports indicated the upregulated expression of PDL1 by dendritic cells of mCRPC patients progressing on AR antagonists. A phase Ia clinical study was initiated to investigate atezolizumab (anti-PDL1), in patients with mCRPC (NCT01375842) [78]. The outcomes showed that atezolizumab exhibited favorable safety with long-term disease control in heavily pretreated mCRPC patients. The combination of abemaciclib with atezolizumab is being tested in an ongoing clinical study in mCRPC patients with CDK12 loss of function mutation [79]. The outcomes are still anticipated.

### Glioblastoma (GBM)

The hallmark of genomic instability in GBM is the perturbation in the cell cycle [80]. Genomic profiling of GBM has characterized alterations in core signaling pathways connected with CDK, such as inactivation of the CDKN2 locus (50%) encoding the CDK4/6 inhibitor p16INK4 and the amplification of CDK4 (13%) and CDK6 (1.5%) [81]. Furthermore, molecular studies have indicated that the CDK4/6-Rb axis is dysregulated in roughly 80% of GBM cases [82]. Therefore, CDK4 and CDK6 are valid targets to be tackled for GBM treatment.

CDK4/6 inhibitors have generally demonstrated positive outcomes in preclinical studies of glioblastoma. However, clinical testing of CDK4/6 inhibitors in glioma patients did not produce promising outcomes [83, 84]. Studies showed that abemaciclib has the capacity to cross the blood–brain barrier achieving cerebrospinal fluid (CSF) concentrations comparable to plasma levels which implies its potential merit for glioma [85]. At present, there are four ongoing clinical trials investigating abemaciclib in patients with GBM (NCT02644460, NCT04074785, NCT04391595, and NCT02981940). INSIGhT phase II trial assessed the efficacy of abemaciclib combination with the standard of care in patients with newly diagnosed GB (NCT02977780). The outcomes showed that abemaciclib enhanced the PFS but not the OS versus the standard of care (radio-chemotherapy). Safety reports indicated that abemaciclib was well tolerated [86]. A phase II clinical trial was initiated to investigate abemaciclib in combination with pembrolizumab in patients with recurrent glioblastoma (NCT04118036). Unfortunately, the trial was halted due to safety issues.

### Colorectal cancer (CRC)

CDK4/6 as well as cyclin D1 are typically overexpressed in CRC and are linked to poor prognosis [87]. The expression of cyclin D1 in patients with CRC helps to predict responses to future CDK4/6 inhibitors [88]. Clinical testing of CDK4/6 inhibitors as monotherapy did not produce any positive responses. Dysregulated expression of CCNE, RB1, AKT1, AURKA, and RAS confers resistance against CDK4/6 inhibitors [89]. As a result, the therapeutic potential of CDK4/6 inhibitors has been assessed in CRC in the form of combination with other agents including Raf, and mitogen-activated protein kinase (MAPK) inhibitors and ICIs [90]. Interestingly, KRAS-mutant CRCs were especially responsive to a combination of MAPK and CDK4/6 inhibitors [91].

There is an ongoing Phase Ib/II clinical trial investigating the safety and efficacy of CDK4/6 inhibitor (SHR6390) combined with the anti-PD-1 antibody camrelizumab (SHR-1210) in patients with advanced CRC, HCC or NSCLC(NCT03601598). The 1st phase of the trial is the dose-finding phase. Then, the trial will proceed for the expansion phase. The outcomes of this study are still awaited to evaluate the safety and efficacy of this combination in CRC.

### Sarcomas

Sarcomas are rare poorly controlled malignant heterogeneous tumors originating from the connective tissues of bone or soft tissues including fat, muscle, blood vessels, or nerves with altered cell cycle and cyclin D-CDK4/6- Rb pathway. Clinical and preclinical studies showed efficacy of CDK4/6 inhibitors in different types of sarcoma[7]. Advanced soft tissue sarcoma overexpressing CDK4 was targeted by palbociclib in a phase II clinical trial, and the results showed median PFS of 4.2 months, and OS of 12 months[92].

Many clinical studies of different phases targeting different types of sarcoma showed that ICIs like pembrolizumab, Ipilimumab, and others showed wide range of PFS and OS due to the heterogeneity of sarcomas. So combination with CD4/6 inhibitors is a good strategy to improve the response[93]. The combination of CDK4/6 inhibitors and immunotherapy against sarcoma is being studied in two ongoing clinical trials. Palbociclib is combined with retifanlimab (anti-PD1) in a phase II study clinical trial in patients with advanced dedifferentiated liposarcoma in which CDK4 gene is highly amplified (NCT04438824)[94]. Palbociclib and pembrolizumab are being investigated in an ongoing phase Ib clinical trial for patients with sarcoma (NCT06113809).

## In silico investigation of CDK4/6 inhibitors, drug targets, and pathways

### In silico methods

The SwissTargetPrediction (http://www.swisstargetprediction.ch/) was used to identify the protein targets of four CDK4/6 inhibitors: abemaciclib, dalpiciclib, palbociclib, and ribociclib. SwissTargetPrediction is a web-based tool that predicts human protein targets for small molecules by analyzing their 2D and 3D structural similarities to known ligands [95]. It requires a SMILES (Simplified Molecular Input Line Entry System) input and provides a ranked list of protein targets with associated probability scores. Predictions with a confidence score of zero were excluded. The predicted targets for all four inhibitors were merged, which served as the basis for subsequent pathway enrichment analysis.

Cytoscape (version 3.9.1) [96], a platform for network visualization and functional analysis, was utilized to explore the biological pathways enriched among the CDK4/6 inhibitor targets and to gain insight into their potential biological regulations. The ReactomeFIViz plugin [97] was used to perform Voronoi tessellation-based FoamTree clustering, which enables system-level interpretation of functionally grouped biological pathways. In addition, Gene Ontology (GO) Biological Processes (BPs) were identified using the g:Profiler web tool (https://biit.cs.ut.ee/gprofiler/gost), which performs functional enrichment analysis by mapping input genes to GO terms and other biological annotations.

To study the protein expression of the identified CDK4/6 inhibitor targets across different tissues and cell types, the Immunohistochemistry (IHC) data from the Human Protein Atlas (HPA, version 24.0) [98] were retrieved. The HPA IHC dataset includes protein profiles from 45 healthy human tissues, determined by immunohistochemistry using tissue microarrays. It contains Ensembl gene IDs, tissue and cell type annotations, expression levels (categorized as “Not detected,” “Low,” “Medium,” or “High”), and measurement reliability (classified as “Approved,” “Enhanced,” “Supported,” or “Uncertain”).

**Fig. 3:**
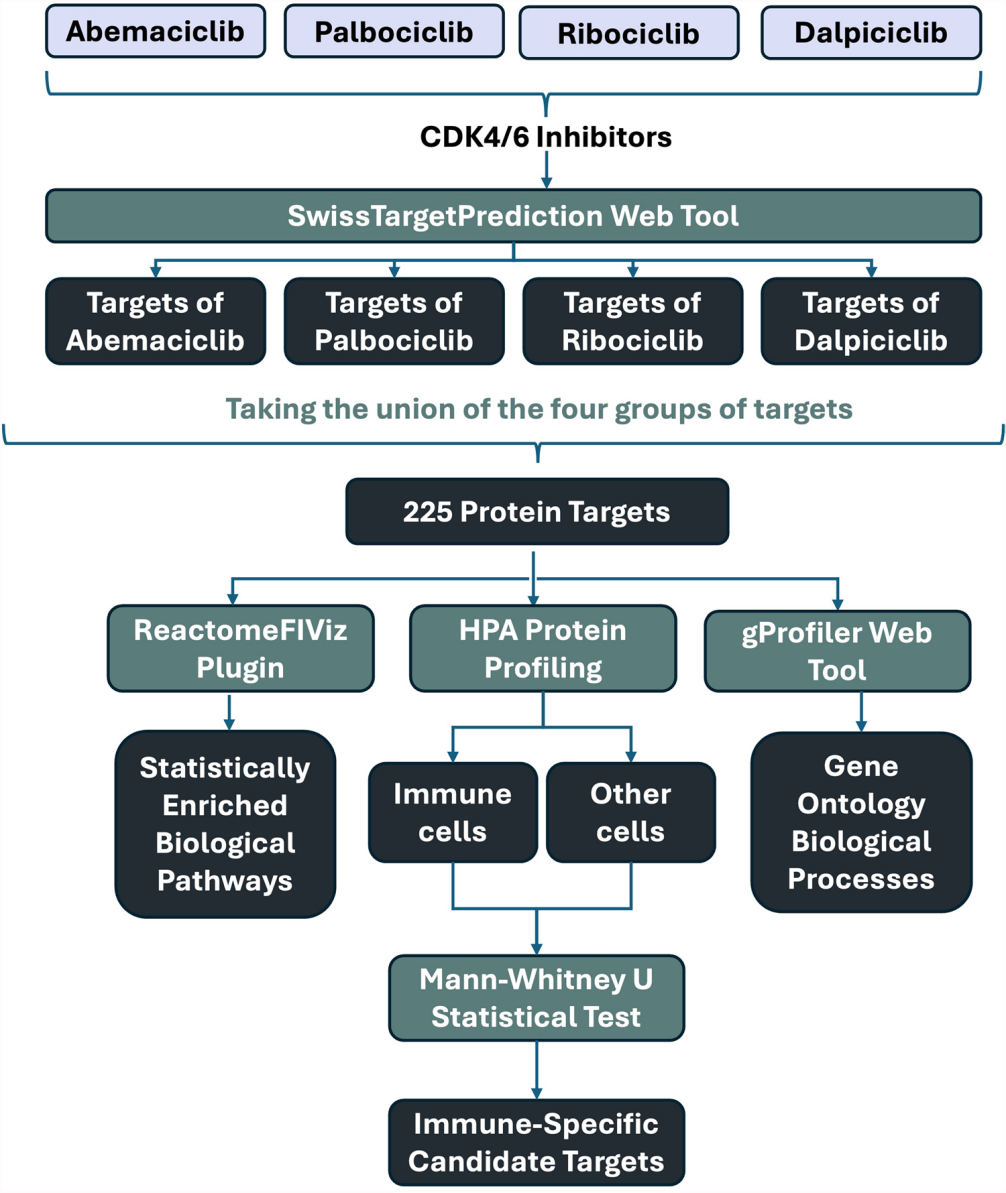
Schematic overview of the methodology for the in silico analysis

Data labeled "Uncertain" were excluded from analysis. For quantitative comparison, expression levels were converted to ordinal values: Not detected = 0, Low = 1, Medium = 2, High = 3. A Mann–Whitney U test was conducted to assess the differences in protein expression between immune and non-immune cell types for each target. Cell type annotation included 127 cell types, including immune cell types such as *lymphoid tissue, macrophages, hematopoietic cells, germinal center cells, non-germinal center cells, Hofbauer cells, Langerhans cells, lymphocytes, mucosal lymphoid cells, Paneth cells, medullary cells, and cells in the red and white pulp*. To correct for multiple comparisons, the Benjamini–Hochberg correction was applied to p values. A schematic summary of the analysis pipeline is provided in Fig. 3.

### In silico results

**Fig. 4:**
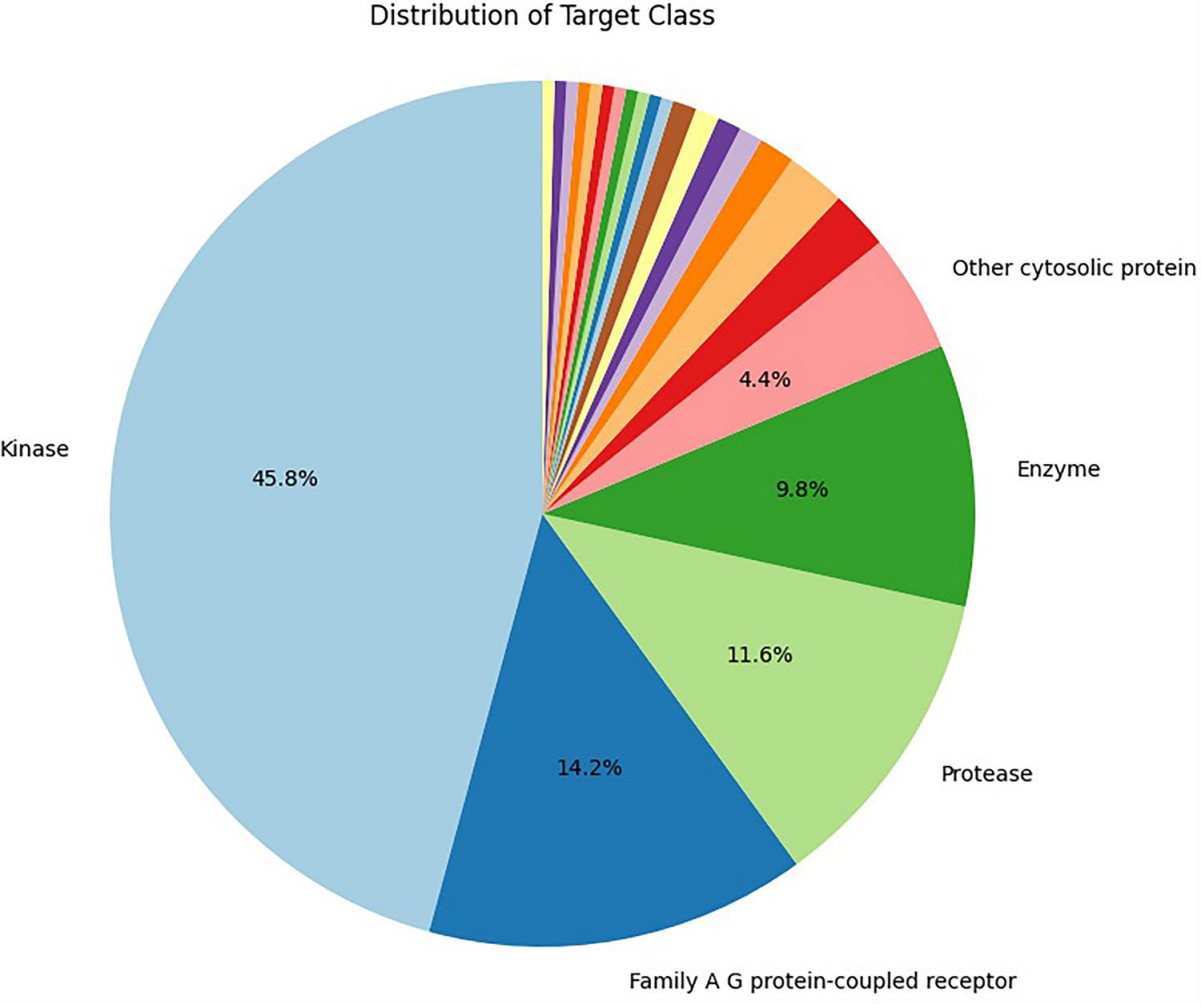
Distribution of protein targets of the four CDK4/6 inhibitors. This diagram demonstrates the categorization of the 225 predicted targets, showing kinases (45.8%), family A G protein-coupled receptors (14.2%), and proteases (11.6%) as the top three categories

**Fig. 5:**
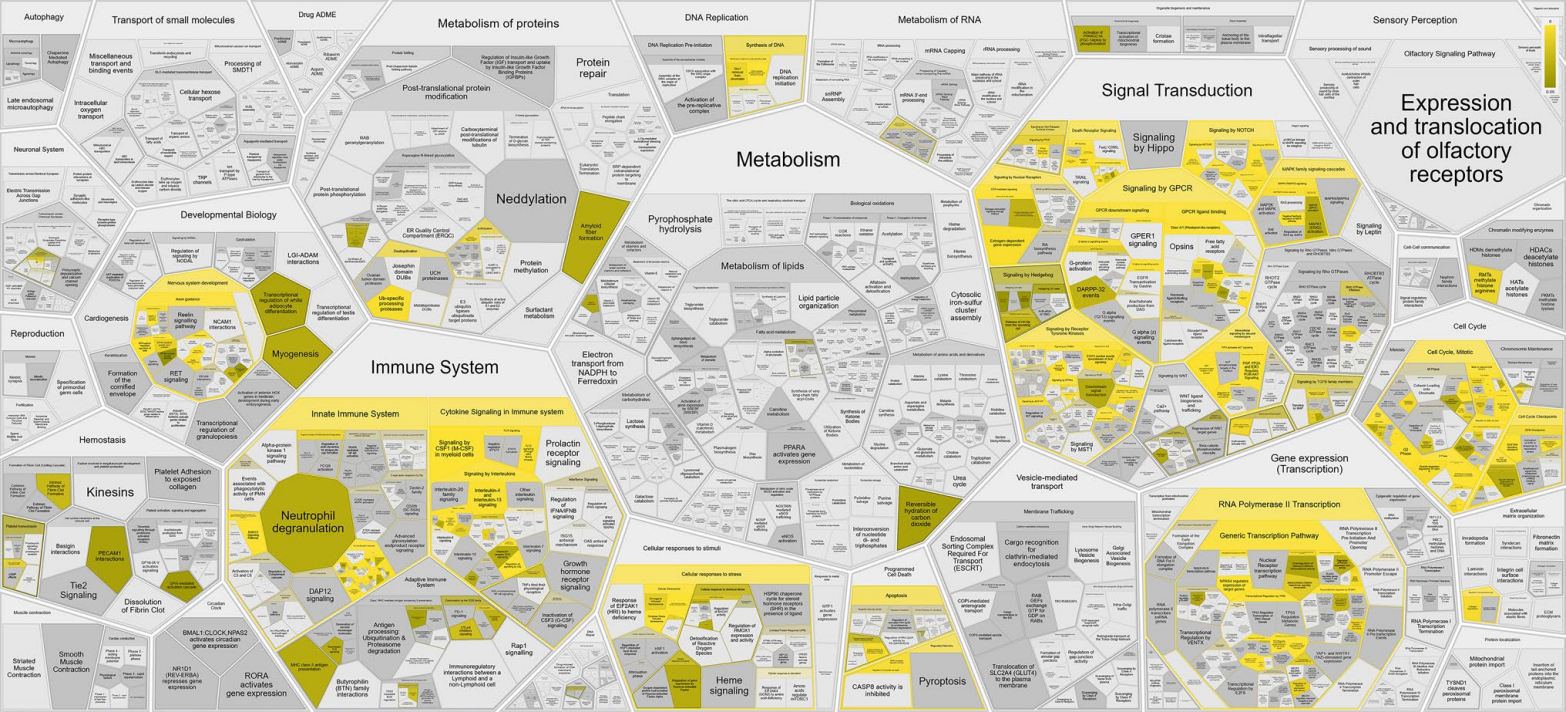
The Voronoi treemap of the significantly enriched Reactome pathways influenced by 225 CDK4/6 inhibitor protein targets

Protein target prediction resulted in a total of 225 protein targets (Supplementary Table S1), of which the top 3 categories are kinases (45.8%), family A G protein-coupled receptors (14.2%), and proteases (11.6%), as shown in Fig. 4. Functional enrichment analysis using the ReactomeFIViz plugin in Cytoscape identified 310 significantly enriched Reactome pathways (FDR < 0.05, Supplementary Table S2), the majority of which were related to signal transduction, cell cycle regulation, and the immune system, as visualized through a Voronoi FoamTree clustering (Fig. 5). These findings highlight the central involvement of CDK4/6 inhibitor targets in core cellular processes as well as immunological functions.

Given the growing interest in combining CDK4/6 inhibitors with immune checkpoint blockades, we focused on exploring the immune-modulatory potential of these targets. Fifty-seven immune-related enriched pathways were identified and categorized into three categories—innate immunity (*e.g., TRIF(TICAM1)-mediated TLR4 signaling and MAP kinase activation*), cytokine signaling (*e.g., Interleukin-17 signaling and FLT3 Signaling*), and adaptive immunity (*e.g., CD28 co-stimulation and CTLA4 inhibitory signaling*). A summary of these pathways is shown in Table 2.

**Table 2:** Summary of Reactome’s immune-related enriched pathways, categorized into three categories: innate immunity, cytokine signaling, and adaptive immunity

Cytokine Signaling			Adaptive Immunity	
Pathway	FDR		Pathway	FDR
Cytokine Signaling in Immune system	5.83E-09		CD28 co-stimulation	0.012
Signaling by Interleukins	6.96E-09		CTLA4 inhibitory signaling	0.02
Interleukin-17 signaling	3.20E-07		Activation of NF-kappaB in B-cells	0.023
Regulation of signaling by CBL	1.75E-04		CD28 dependent PI3K/Akt signaling	0.029
Signaling by CSF1 (M-CSF) in myeloid cells	1.97E-04		Downstream TCR signaling	0.034
FLT3 Signaling	5.28E-04		Costimulation by the CD28 family	0.034
Interleukin-3, Interleukin-5 and GM-CSF signaling	9.97E-04		MHC class II antigen presentation	0.034
FLT3 signaling through SRC family kinases	1.15E-03		Antigen processing-Cross presentation	0.034
Interleukin-4 and Interleukin-13 signaling	1.36E-03		Downstream signaling events of B Cell Receptor (BCR)	0.034
Interleukin-1 signaling	5.07E-03		Endosomal/Vacuolar pathway	0.04
Fcgamma receptor (FCGR) dependent phagocytosis	0.011			
Interleukin-1 family signaling	0.021			
TNFR2 non-canonical NF-kB pathway	0.032			
Interleukin receptor SHC signaling	0.034			
Innate Immunity				
Pathway		FDR	Pathway	FDR
TRIF(TICAM1)-mediated TLR4 signaling		8.80E-09	CREB phosphorylation	8.80E-05
MAP kinase activation		7.94E-08	MAPK targets/ Nuclear events mediated by MAP kinases	1.97E-04
Toll Like Receptor 4 (TLR4) Cascade		3.23E-07	activated TAK1 mediates p38 MAPK activation	2.26E-04
TRAF6 mediated induction of NFkB and MAP kinases upon TLR7/8 or 9 activation		1.32E-06	NOD1/2 Signaling Pathway	2.64E-04
MyD88 dependent cascade initiated on endosome		1.41E-06	ERK/MAPK targets	3.94E-04
Toll Like Receptor 7/8 (TLR7/8) Cascade		1.50E-06	TICAM1, RIP1-mediated IKK complex recruitment	2.13E-03
Toll Like Receptor 9 (TLR9) Cascade		2.07E-06	Nucleotide-binding domain, leucine rich repeat containing receptor (NLR) signaling pathways	3.24E-03
Innate Immune System		3.79E-06	IKK complex recruitment mediated by RIP1	3.46E-03
MyD88 cascade initiated on plasma membrane		4.46E-06	MAP3K8 (TPL2)-dependent MAPK1/3 activation	0.011
Toll Like Receptor 10 (TLR10) Cascade		4.46E-06	TRAF6 mediated IRF7 activation in TLR7/8 or 9 signaling	0.034
Toll Like Receptor 5 (TLR5) Cascade		4.46E-06	Neutrophil degranulation	0.034
Toll Like Receptor 2 (TLR2) Cascade		1.87E-05	IRAK1 recruits IKK complex	0.034
MyD88:MAL(TIRAP) cascade initiated on plasma membrane		1.87E-05	IRAK1 recruits IKK complex upon TLR7/8 or 9 stimulation	0.034
Toll Like Receptor TLR6:TLR2 Cascade		1.87E-05	Activation of the AP-1 family of transcription factors	0.034
Trafficking and processing of endosomal TLR		5.09E-05		

Several enriched immune pathways have been previously identified through experimental studies. For example, signaling by colony-stimulating factor (CSF1) in myeloid cells (FDR = 1.97E-04) and granulocyte–macrophage colony-stimulating factor (GM-CSF) (FDR = 9.97E-04) were enriched, consistent with the findings of Kumar et al. [99], who showed that CDK4/6 inhibition enhances CSF2 expression during abemaciclib treatment, promoting anti-tumorigenic M1 macrophage polarization and enhancing antigen presentation. In addition, multiple MyD88-related pathways were enriched, supporting prior evidence that CDK4/6 inhibitors can reprogram the tumor microenvironment by modulating the IL-33–MyD88 axis, therefore inhibiting suppressive immune cells [100]. Furthermore, Gene Ontology (GO) Biological Process (BP) enrichment analysis further supported these findings, yielding 1,613 enriched terms (FDR < 0.05, Supplementary Table S3). Among these were several strongly immune-related processes, including positive regulation of the immune system process (GO:0002684 with FDR of 8.24E-15), immune effector process (GO:0002252 with FDR of 4.97E-06), and immune response-regulating cell surface receptor signaling pathway (GO:0002768 with FDR of 4.00E-08).

To further investigate the relevance of these targets in different cell types, we analyzed the protein expression profiles of the 225 predicted targets using the HPA IHC dataset. This analysis showed that 24 protein targets exhibited a statistically significant higher expression in immune cell types (adjusted *p <* 0.05). In comparison, four targets showed higher expression in non-immune cell types. These include targets such as HLA-DRB1 (adj p-val 2.00E-13), JAK3 (adj p-val 3.46E-09), and TLR7 (adj p-val 1.90E-08). Identified proteins may serve as context-specific targets through which CDK4/6 inhibitors exert immunomodulatory effects. Their immune-preferential expression profiles may help guide further mechanistic or biomarker-driven studies **(**Supplementary Table S4**)**.

Together, these results suggest that the targets of CDK4/6 inhibitors are not only involved in classical cell cycle pathways but also participate in key immune system processes. The functional enrichment and cell type-specific expression analyses together support a model in which CDK4/6 inhibition could synergize with immune checkpoint blockade by affecting immune signaling networks.

## Conclusion and future perspectives

The studies depicted in this review highlight the valuable potential of CDK4/6 inhibitors to sensitize various solid malignancies to immune checkpoint inhibitor (ICI) immunotherapy. The review encompasses numerous solid tumor types for which clinical data have been published or ongoing clinical studies are underway. Early clinical data suggest that combining CDK4/6 inhibitors with immune checkpoint inhibitors (ICIs) holds therapeutic promise across multiple breast cancer subtypes, including hormone receptor-positive/HER2-negative (HR + /HER2 −) and androgen receptor-positive triple-negative breast cancer (AR + TNBC). Despite that, a significant challenge observed across several clinical trials is the heightened incidence of severe adverse events (AEs) when CDK4/6 inhibitors are combined with ICIs, particularly abemaciclib. Combinations like abemaciclib/pembrolizumab ± anastrozole in HR + /HER2- metastatic breast cancer led to two deaths and a high incidence of grade ≥ 3 AEs, including neutropenia, diarrhea, and liver function alterations, leading to trial discontinuation. Similar safety concerns prompted the termination of abemaciclib/nivolumab studies in HNSCC, NSCLC, HCC, and recurrent glioblastoma. High-grade hepatotoxicity was also reported with palbociclib/nivolumab/ anastrozole in primary breast cancer, causing study termination. These findings imply that such combinations may augment treatment-related toxicities. The high incidence of toxicity in some combination regimens highlights the critical need for better dosing optimization to improve tolerability and maximize the benefit/risk ratio. This includes exploring different schedules and drug sequences. The data from clinical trials in ovarian cancer, prostate cancer, and CRC are still awaited to judge if these novel approaches will allow the broader utilization of checkpoint inhibition in such settings. Efforts are needed to identify reliable biomarkers that can predict which patients or tumor types will best respond to CDK4/6 inhibitor combinations, especially with ICIs, and to help anticipate and manage toxicities. The identification of immune-preferential protein targets (e.g., HLA-DRB1, JAK3, TLR7) through in silico analysis offers a promising avenue for guiding further mechanistic or biomarker-driven studies.

## Supplementary Information

Below is the link to the electronic supplementary material.

Supplementary file1 (XLSX 22 KB)

Supplementary file2 (XLSX 33 KB)

Supplementary file3 (XLSX 150 KB)

Supplementary file4 (XLSX 23 KB)

## Data Availability

No datasets were generated or analyzed during the current study.
